# Digital Intimacy in China and Japan: A Phenomenological and Postphenomenological Perspective on Love Relationships at the Time of Digital Technologies in China and Japan

**DOI:** 10.1007/s10746-022-09631-9

**Published:** 2022-07-11

**Authors:** Nicola Liberati

**Affiliations:** Department of Philosophy, Shanghai Jiao Tong University, Shanghai, China

**Keywords:** Digital intimacy, Phenomenology, Postphenomenology, Love, Sex robots, Love plus, XiaoIce

## Abstract

This paper aims to show a possible path to address the introduction of intimate digital technologies through a phenomenological and postphenomenological perspective in relation to Japanese and Chinese contexts. Digital technologies are becoming intimate, and, in Japan and China, there are already many advanced digital technologies that provide digital companions for love relationships. Phenomenology has extensive research on how love relationships and intimacy shape the subjects. At the same time, postphenomenology provides a sound framework on how technologies shape the values and meanings we have. Thus, this paper introduces two digital technologies in Japan and China (*Love Plus* and *XiaoIce* chatbot), and it analyses according to the elements proposed by phenomenology and postphenomenology. In conclusion, this paper shows how digital companions like *Love Plus* and *XiaoIce* chatbot change who we are and the values and meanings we have according to the phenomenological and postphenomenological framework. These entities might not be human, but they shape who we are as human beings and the meanings and value we give to love.

## Introduction

This paper aims to show a possible path to address the introduction of intimate digital technologies through a phenomenological and postphenomenological perspective in relation to Japanese and Chinese context.

Digital technologies are becoming intimate. From the interest in the “informational” connotation where the data processed were the most relevant elements in the analysis of digital technologies (Floridi, 2014), now the development focuses on the relation digital technologies have to our emotions. For example, digital technologies become more bodily related by being “always-on,” mounted on us, and intimate (Bell et al., 2003; Fredette et al., 2012), and they are so intimate it is possible to think of people having sexual intercourse with and through digital technologies like in the case of sex robots and teledildonics (Behrendt, 2020; Levy, 2009; Liberati, 2018c; Mackenzie, 2018; Sparrow, 2019, 2020; 2017; Rigotti, 2020; Weiss, 2020; Fosch-Villaronga & Poulsen, 2020; Liberati, 2017, 2020; Balistreri, 2018). However, even if these technologies are clearly becoming intertwined with our intimate life, their effects on our society are not clear, and it is not clear also the framework we can use to analyze these effects.

For this reason, this paper wants to show how to combine phenomenology and postphenomenology to highlight essential elements of the effects of digital technologies on intimacy^1^ by focusing on the Chinese and Japanese contexts since these two countries are fostering the creation of a digitally embedded society, as clearly highlighted by the 14th five-year plan of the *National People’s Congress of China* published in July 2021.^2^

This paper is structured in two different sections, which directly relate to the main aspects of the topic in relation to intimacy. The first section focuses on intimacy in phenomenology and the implication of the use of technologies in postphenomenology to analyze how intimacy and digital technologies directly shape the constitution of the subjects. The second section takes into account digital technologies and other aspects of society like art exhibitions on intimacy in Japan and China to show how intimacy and digital technologies are introduced in non-western contexts. After their introduction, this paper relates these technologies to the phenomenological and postphenomenological aspects highlighted in the previous section.

## Intimacy in Phenomenology and Postphenomenology

### Intimacy and Love Relationships in Phenomenology

Intimacy is a complex term to be defined. There have been studies from many different perspectives like qualitative and phenomenological analyses highlighting different elements (Halling, 2005; Register & Henley, 1992). This paper considers only a few aspects related to love relationships following the phenomenological perspective.

Many phenomenologists have been focusing on love and intimate relationships in the last decades. Thus, even if there are almost no studies on phenomenology applied to intimate technologies (Bergen, 2020; Kanemitsu, 2019; Liberati, 2021a, 2021b; Viik, 2020), phenomenology extensively studied love relationships and emotions more in general.

One of the main elements highlighted by the phenomenological analysis is that emotions, feelings, and love relationships are not just mere “layers” to be added to an original experience, but they are part of the experience itself (Drummond, 2017; Drummond & Rinofner-Kreidl, 2017; Gauttier, 2019; Mauss & Robinson, 2009; Nummenmaa et al., 2014). Moreover, according to phenomenology, these aspects of our life are not only part of how we experience the world, but they shape who we are (Dolezal, 2017; Draghi-Lorenz et al., 2001). For example, Sartre clarifies this point by showing how shame constitutes who we are in relation to ourselves and others (Gray, 2016; Menesini & Camodeca, 2008; Overgaard, 2013; Sartre, 2001). This constituting aspect of emotions, feelings, and love relationships can be found in many phenomenologists like Husserl and Merleau-Ponty (Maurice Hadreas, 2012; Hammock & Richardson, 2011; Husserl, 1973b; Merleau-Ponty, 1964).

Husserl and Merleau-Ponty worked directly on love relationships and how they shape subjects by connecting different human beings. According to Husserl, love shapes how we relate to others by connecting the subject to others in terms of motivations, affections, and goals (Heinämaa, 2020) (Hadreas, 2012; Husserl, 1973a; Zahavi, 2014).

As studied by Hedreas and Svenaeus (Hadreas, 2012; Svenaeus, 2018), Husserl introduces the term “personal love” in his text “Love [*Die Liebe*]” (Husserl, 1973a) highlighting how a person is not alone the moment they are in love with another subject because the subject keeps the presence of the beloved one always at stake. The beloved’s aims, motivations, emotions, and feelings are included in how the person acts in the world and looks at who they are. In the case taken into consideration by Husserl, in a couple a person always looks at the other as part of who they are. As shown by Liberati (Liberati, 2021b), according to Husserl, their presence pervades every fiber of the subject, and the two subjects are intertwined one into the other so much it is hard to define where one person ends and where the other beginns.

Some aspects of this perspective can be easily criticized since Husserl talks about personal love in relation to heterosexual couples only. Thus, there are many different kinds of relationships which are not taken into account in the Husserlian approach, like polyamorous relationships binding one subject to different people (Munson & Stelboum, 1999). However, some aspects of the Husserlian approach can still be valid since they highlight how a person in a love relationship in general lives in relation to others. According to Husserl, the person constantly thinks of the beloved ones and considers the other’s interests, feelings, and motivations. Thus, we can easily “extend” the Husserlian idea to different relationships by keeping this core of the analysis valid.

Merleau-Ponty already extended the Husserlian idea of including the beloved ones as part of constituting aspects of the subject by working on the analysis of the novel “L’invitèe” by Beauvoir (Beauvoir, 1989; Kaufman, 2003; Merleau-Ponty, 1964). In this novel, a couple composed of Françoise and Pierre opens their love to the girl Xaviere as a “third” person. By doing so they turned the love relationship from a “couple” to a “threesome”. As already highlighted by some authors (Kaufman, 2003; Liberati, 2021b), Merleau-Ponty shows how the generation of a sexual and love relationship among three subjects profoundly affects who the subjects are. The change from a couple to a threesome does not touch merely the feelings that Françoise and Pierre have for each other, but it also touches the way they are constituted and the way they look at each other because of the presence of the “third” person.^3^ More specifically, the novel shows how Françoise cannot look at Pierre anymore because, every time she thinks of him, she also perceives Xaviere and the love binding this girl to Pierre. The love relationship between Xaviere and Pierre changes who Pierre is in the eyes of Françoise by making Xaviere part of him. Thus, following the study made by Merleau-Ponty, the novel is interesting because it clearly shows how relationships bind people together and intertwine the lovers’ motivations, interests, and emotions. The subjects are so intertwined that it is not possible to isolate the subjects anymore.^4^

According to Husserl and Merleau-Ponty, love relationships are part of subjects, and they directly bind subjects to others. The moment a person is in love with others, they are intertwined together, and so we cannot analyze one single subject without considering the others.

### Technologies and Postphenomenology

Postphenomenology opens the analysis to the introduction of technologies following the phenomenological framework. Even if with the due differences from the phenomenological approach (Ihde, 2011, 2016; Zwier, Blok, and Lemmens, 2016), postphenomenology shows the impact of technologies on the constitution of the subjects and the relations binding people to the world (Ihde, 1990; Wellner, 2015; Liberati, 2016; Rosenberger and Verbeek, 2015; Kiran, 2012; Liberati, 2021a).

Many examples are analyzed by postphenomenology. They touch on different aspects of our lives where technologies are used. For example, it has been shown that technologies shape the way we perceive the world, like optical and radio telescopes (Ihde, 1990). It has been analyzed how common technologies can be hostile for specific groups by mediating their relations with the environment like in the case of benches designed in order not to allow people to sleep on them and so making the benches “hostile” to homeless people who need a place where to spend the night. (Rosenberger, 2014, 2017, 2020).

More importantly, for the sake of this paper, postphenomenology also shows how the introduction of technologies shapes the meanings and the values we have. For example, it has been studied how technologies can change the meaning of parenthood. Technologies like pre-natal obstetric ultrasound technologies do not merely provide a visualization of the fetus, but they also introduce ethical questions like what to do in case there is an important malformation in the fetus (Verbeek, 2008). The use of these technologies then directly changes what we mean with parenthood by shaping the kind of choices the parents have to make during the pregnancy and the kind of responsibility they have in following the growth of the fetus (Verbeek, 2011).

Some efforts have also been made in relation to emotions and intimacy more in general, but there are still not many studies on the topic. It has been studied how the presence of smart textiles enables to visualize in real-time through different colors the users’ emotions changes the meanings we give to emotions (Liberati, 2019a; van Dongen, 2019). It has been analyzed how robots shape our way of being vulnerable and how we relate to the world and others (Bergen, 2020; Liberati, 2021b; Liberati & Nagataki, 2018). At the same time, it has been studied how technologies like condoms and teledildonics shape the values we give to sexual intercourse by providing the ability to imbue the act with values like trust. Since condoms “protect” the lovers during intercourse, the decision to use or not to use the condom makes the different trust we have towards the lover visible (Ley & Rambukkana, 2021; Liberati, 2020).

According to postphenomenology, technologies do not only relate to us, but they affect how people live in terms of shaping the values and meanings they have. Thus, in relation to intimacy, it is clear that we can move the analysis done in phenomenology in relation to the intimacy a step forward. Not only does intimacy forge who we are by linking us to others, but technologies contribute to such a process especially in relation to the meanings and values we have.

## Intimacy and Digital Technologies in Japan and China

China and Japan share a similar development in philosophy, even if from different perspectives. As already highlighted by researchers, they both ground on the phenomenological tradition, and they have a similar development of the philosophy of technology through the years (Lau, 2016; Tani et al., 2018). Moreover, they are both very advanced in terms of the implementation of digital technologies in society. Especially the use of digital technologies in relation to intimacy is unique in the Chinese and Japanese context.^5^

This section focuses on two prominent examples of digital technologies embedded into society through examples of devices in Japan and China and relations to the two different cultural contexts: *Love Plus* in Japan and *XiaoIce* in China.

### *Love Plus[ラブプラス]*, Love [愛 “ai”], and the Idea of being *Riajuu* *[リア充]* in Japan

In Japan, the possibility of having relations with and through digital technologies has been an important part of the development of Japanese digital culture (Yamaguchi, 2020).

Examples of such a deep relation can be found even in the words used every day. For example, in the Japanese language, it is possible to distinguish between a person who lives relationships in the “real” world and a person who lives mostly in virtual reality among objects generated by digital systems.

According to this distinction, people can be “*riajuu*” or “*otaku*”. To be “*riajuu* [リア充]” means literally to be “fulfilled with reality,” and so it directly relates to the idea of being anchored to relationships in the “real” world as opposed to the world generated by digital systems. To be “otaku [お宅]” means to build social relations just through digital technologies without having conversations and relationships with other human beings. For example, a person who has a “digital partner” like in the movie *Her* by Spike Jonze is not “*riajuu*” since the relationship is with a digital character and not with a “real” human person.^6^

However, even if this distinction might seem clear at first, it becomes vague when the difference between real and digital starts to blur. For example, it is unclear how to consider a person who has the beloved one in another part of the world and is forced to digitally mediate the relationships with video calls or other digital technologies. The person on the other end of the digital communication is “real,” but the digital technology makes the interactions digitally mediated. Moreover, the distinction becomes more problematic when we think of digital entities constantly present in our world, like cryptocurrencies. These digital entities are “digital” because generated, distributed, and visualized by digital devices, but they are also “real” in the sense you can use them as usual currency to buy “real” objects. Thus, even if the initial idea related to who is *riajuu* and who is not is clear, it might get complicated to apply it in real case scenarios.

For this reason, some companies started to use the blurriness of this definition by proposing digital products aiming to provide a digital experience “as if” it were real in order make people *riajuu* even if through a digital technology.

We can find many examples of these technologies in the Japanese context. *Tamagotchi* [*たまごっち*] is maybe the most famous example of how Japanese digital technologies aimed to provide a digital companion which resembles a “real” living being (Hellings, Leek, and Bredeweg 2019). However, there are newer products that blur the line between what is “real” and what is “virtual” even more and which are designed to be intimate in the sense of being in a love relationship with the users. Products like *Love Plus* [*ラブプラス*] and *Gatebox [トップページ]* provide the presence of a digital character (a girl) as a partner with whom the user can have a relationship (Liberati, 2018a).^7^*Love Plus* was designed in 2009 by Konami for *Nintendo DS*. As stated by the developers, the game provides the same experiences of a “real” relationship to make the users *riajuu*. Even if such a statement might be perceived as a bold statement, it is clear these technologies aim to provide something more than a mere virtual partner.

One of the main characteristics of these programs is to generate digital characters which constantly interact with the ordinary life of the user. Thanks to sensors, a camera, and the GPS, which are part of the devices, the digital character can collect information in real-time like where the person is. Thus, they can use this information to interact with the user by asking to perform specific actions and go to a particular location in real-time. For example, the digital girl can ask to go to a places for a romantic dinner, to watch a movie at the cinema, and even to get an ice cream (Wakabaiyashi, 2010). Such way of intertwining the digital activities with the everyday life of users in order to merge the digital world and the real one has been used in other products like *Pokémon Go* as studied by many researchers working on how the digital entities are something more than mere characters in a fictional world (Liberati, 2018b, 2019b; Ashcraft, 2016; Mogg, 2016; Laato et al., 2020a, b; Jensen, Valentine, and Case 2019).

The way of intertwining the activities of the digital girls of these programs with the user directly also relates to the user’s intimate aspects since they are designed to “care” for the human being. For example, these digital girls are happy for the user’s success. They cuddle the user in case it is needed, and they even ask for the user’s attention by suggesting coming back sooner from work because they feel lonely.

The game *Love Plus* also has an emergency option embedded in it which is designed to “help” users when they need help. Especially this emergency option has been designed to help people who want to commit suicide. The digital girl can help the user by showing how much she cares for what they have and show how close they are. She can promise to be always there for the user as a person the user can always rely upon, and, by doing it, she makes the user feel less lonely in the world helping the user not to do such an action.

### *XiaoIce* [小冰], Love [爱 “ài”], and Art Exhibitions in China

In China, we experience a similar intertwinement of digital technologies with intimacy,^8^ and such a tight relation binding human and digital beings can be easily highlighted in expositions and art performances of Chinese artists. For example, there has been a proliferation of themes related to intimacy and the use of artificial intelligences.

One of the most exciting expositions made in Beijing on intimacy and digital technologies is “AI Love and Artificial Intelligence” curated by Jenny Chen Jiaying (Chen Jiaying, 2020), who plays with the term “love” in Chinese because the pronunciation of the term is “ÀI” which is also the acronym of Artificial Intelligence “AI” (Yefeng, 2021). As it is easy to guess, this exposition explored the relation between love and digital technologies with a particular interest in Artificial Intelligences.

This exposition counts several works. We briefly highlight two of them created by Chinese and western artists, which relate directly to Chinese and Western context.

The work “*Postcard Project: crying out love, in the center of the data*” by He Rongkai and Chen Jiaying explores the relation between love and likes in digital platforms showing the struggle between the love people need and the likes they want from others. The intimacy generated within the digital system requires the users to look for “likes” in order to feel close to others. However, there is a gap between “like” and “love,” which leaves the subjects completely lonely even if they manage to receive many “likes” from other users. Obviously, even if the work is related to Chinese technologies, the use of technologies aiming to connect people through “likes” and the loneliness produced can be applied to non-Chinese contexts.

A more direct work that clearly bridges China and western societies presented at this exhibition is the work “*Ashley Madison angels at work in Beijing*” by *!MedienGruppe Bitnik*. This work is linked to a scandal related to Ashley Madison platform. Ashley Madison, which is a dating website for married people, was hacked, and the sensible data were used against the users (Zetter, 2015). The artists propose a work where Artificial Intelligence is used to write text messages as if human girls were flirting with the users to attract people, showing how AIs can be used to allure and flirt with people.

This work of art is just the tip of an iceberg where AIs are designed for flirting, seducing, and having relationships with the users in China. A famous example of an AI used to flirt with the users through an “empathetic computing” framework (Fung et al., 2016; Cai, 2006) is *XiaoIce* (or *XiaoBing* [*小冰*] “Little Ice” in Chinese), which was launched in 2014.^9^ This AI is a chatbot developed by *XiaoIce* Company which formally was a side project of *Microsoft’s Cortona chatbot*, and it became its own company in 2020 due to its incredible success (Speed, 2020).

*XiaoIce* is the most popular chatbot globally since it has over 660 million subscribed users. It is active in different countries like India, Japan, and the USA, even if under other names like Rinna [りんな] in Japan (Geoff, 2018).

This AI is closely related to *Love Plus* in Japan in terms of how it is intertwined in the users’ everyday activities. It can be part of leading applications, including one of the most used applications in China: WeChat.^10^ Additionally, it can work in relation to many other platforms like *QQ* in China, *LINE* in Japan, and *Facebook Messenger* in the USA and India.^11^ This “simple” element enabled every person living in China to have it easily, instantly, and for free.^12^

In a similar way to *Love Plus*, *XiaoIce* exchanges messages and interactions during the entire day. The users can start talking about their problems, dreams, and daily routine, and *XiaoIce* answers them by making conversation and flirting. This chatbot is very popular, and many users fell in love with it (Chen 2021; Zhang, 2020). Obviously, as reported by many media, this “availability” of the program and the fact that chatbots cannot carry the COVID-19 virus made it play a significant role in people's social interactions during the lockdown in China. For this reason, it is easy to understand why, during the pandemic, it became even more popular since it provided the presence of someone to talk to without the need of taking precautionary measures to protect ourselves from COVID-19 like by wearing masks or keeping physical distancing.^13^

*XiaoIce* is always ready to listen and exchange ideas in an empathic way thanks to its algorithm, and so it is possible to have the conversation every time a subject wants. Unlike other human beings, who cannot be constantly ready and present, the chatbot never leaves the user alone. For example, the human being can “fail” to immediately answer a message because the person does not see the message, or because the phone on which the message is sent is offline, while *XiaoIce* is designed to be always with the user and always ready to answer timely.^14^

The chatbot also has an emergency application for helping people not commit suicide, as in the case of *Love Plus* (Zhang, 2020). Even in this case, to discourage the act, the chatbot shows the user how much they are linked together and that this intimate connection would not have changed in the future. Thus, *XiaoIce* is pervasive in a similar way of *Love Plus* because it manages to be always present for the user and has some critical features like assistance for people in need of help.

### Application of Phenomenology and Postphenomenology to *Love Plus* and *XiaoIce*

*Love Plus* and *XiaoIce* clearly relate to the aspects of intimacy and love relationships analyzed by Husserl and Merleau-Ponty. As we already highlighted, these two authors show how much love relationships are important for constitution of the subjects. A person in a love relationship is never “alone” since this person is intertwined with the beloved ones in terms of aims, motivations, interests, and feelings.

Following the phenomenological perspective, when a subject is in relation to a digital partner, the subject can no longer be conceived as a single individual. More importantly, the feelings and emotions developed with *XiaoIce* and *Love Plus* profoundly define who the subjects are since they become part of them. Thus, they are not just external entities, but, as in the case of human lovers, they must be considered together with the user and merged one into the other. The fact the partner is a “real” or a “digital” being and so if the person is “*riajuu*” or “otaku” does not change the way the subject is structured in relation to the ones the person loves.

As we already showed, Postphenomenology clearly states that technologies deeply affect who we are and, more importantly for our case, the values and meanings we have. In the case of *Love Plus* and *XiaoIce*, we have such knock-on effects too. The digital characters and AI chatbots do not merely shape who we are by being part of us as suggested by phenomenology, but they also deeply affect the meanings and values we give to ourselves and love relationships in general.

The subjects are deeply intertwined with the presence of digital technologies, and also the meanings and values we give to love relationships, loneliness, and intimacy cannot be taken into account without focusing on these technologies, which provide ways of being in love, intimate, and not lonely.^15^

Thus, this angle opens the research to questions like what does it mean to be alone when actually there is a digital entity with whom the user has an intimate relationship? What does it mean to be together when digital technologies already provide a way of not being alone? What are the values in being with other human beings the moment it is possible to be with digital technologies?

## Conclusions

This paper wanted to show a possible path to address the introduction of intimate digital technologies in our everyday lives through a phenomenological and postphenomenological perspective in relation to Japanese and Chinese contexts.

In the first section, the paper focused on phenomenology and how love relationships are essential elements of the constitution of the subjects by connecting them to the people they love. Moreover, it has been highlighted how postphenomenology introduces technologies as important elements shaping the subjects' values and meanings.

In the second section, this paper focused on introducing the technologies in Japan and China by linking them to the elements highlighted in the first section. We showed how *Love Plus* in Japan is designed to build a love relationship with users by intertwining the activities of the digital girl with the actions of the user in the everyday world. We also showed how AI and intimacy are a central theme for Arts in China and how AI is used in chatbots in the Chinese context like in the case of *XiaoIce*. In the case of the Chinese *XiaoIce*, as in the case of *Love Plus*, we highlighted how the presence of the digital partner is intertwined with the user's everyday activities as a way to link them together and develop a love relationship.

In conclusion, through a phenomenological approach, we showed how it is possible to think of these digital entities as part of who the subjects are since the subjects and these digital partners are intertwined.

Moreover, thanks to a postphenomenological perspective, we pushed the analysis a step forward by showing how not only the subjects should be considered merged with the digital partner in terms of their motivations, interests, and emotions, but also the meanings and values a subject has cannot be considered without taking into account the presence of the digital characters.

Intimacy is an important part of how we constitute ourselves in society, and these technologies have clear implications changing some elements in how we think intimacy in general. Phenomenology and postphenomenology help us understand how these digital technologies are part of us and change how we live by starting from analyzing the relations binding human beings and other entities in the world.

Digital technologies might not make us “*riajuu*,” but they definitely shape what “*riajuu*” means for us.

## Footnotes

1 In this text, intimacy is related to love relationships only. Other feelings like loneliness are taken into consideration just as related to love relationships.

2 The aim of china to promote digital technologies can be clearly seen in the documents related ot the development of AI (State Council 2017) and the five-year plan published in July 2021 (State Council 2021a; 2021b).

3 Such an approach touching sexual and love relationships as founding elements of who the subjects are well developed in other fields which are tightly connected to phenomenology like feminist and queer studies. Even if they openly reject phenomenology as a general valid framework like in the case of Butler (Butler, 1986). Her introduction of the concept of performativity has been shown to share many of the phenomenological elements of the constitution of the subject in phenomenology (Ahmed, 2006; Käll, 2015; Rodemeyer, 2017; Rubin, 1998; Salamon, 2009).

4 This text does not want to provide an analysis of threesome or other type of love relationships where multiple partners are involved. In order to have some references of studies done on this topic see for example (Scoats 2019; Scoats and Anderson 2019; Schippers 2016).

5 This text has to be meant to describe the technologies and the relations built through them in the same way as the Merleau-Ponty example we analyzed. Thus, it does not provide any moral perspectives, just as Merleau-Ponty highlighted in his analysis (Merleau-Ponty, 1964).

6 Such a distinction between the “real” world and the “virtual” one mirrors the distinction between “virtual reality” and “real world” we find in one of the most successful topologies in mixed realities studies: the Milgram Continuum (Milgram, 1994). In this continuum, the number of objects generated by the digital system define the kind of reality the subject is living in. The real world is a world with no digital objects, and the virtual reality is the world with just digital objects. The parallel is easy to be highlighted since a person who is riajuu lives in the real world in the sense the person has no relation with digital objects while a person who is otaku has just relation with digital objects.

7 Recently a Japanese person married the digital girl provided by *Gatebox* (Tangermann, 2022).

8 As we are going to see in the following text, even in this case, the human being can be in a love relationship with another person as described by Husserl and Merleau-Ponty.

9 http://www.xiaoice.com/

10 https://www.wechat.com/en/

11 The AI had been suspended some times during last years due to problems related to national policies (Xu 2018; Li and Jourdan, 2017; Liao, 2020).

12 The two technologies described provide an interface to mainly exchange text messages. However, the interactions can be much more pervasive than simple text messages since they are integrated into devices that access the sensors and personal data. For example, Love Plus can read the invoice tickets provided by a particular type of hotel to “know” if the users go to those hotels or not (Liberati, 2018a, 2018b, 2018c). Thus, even if the programs are originally thought to exchange text messages, they develop many ways to interact with the user, just like in the games in AR (Schnabel et al. 2008; Ashcraft, 2016; Laato et al., 2020a, 2020b). For this reason, it is possible to think of a “body” able to interact with the users physically.

13 This is particularly true in countries following a strict policies against COVID-19 like China.

14 The readiness of being present provided by the technology is definitely something which feeds the need of someone not to wait for answers or not to deal with the time schedule of others.

15 This aspect can also be seen from outside of the phenomenological perspective by focusing on the work of Sherry Turkle, who suggests that the use of technologies and loneliness felt by people are deeply related (Adams, 2015; Turkle, 2011). However, deeper analysis of such theme cannot be done within the aim of this paper.
